# Fish occurrence in the Kuban River Basin (Russia)

**DOI:** 10.3897/BDJ.9.e76701

**Published:** 2021-12-06

**Authors:** Oleg Artaev, Andrey Pashkov, Dmitriy Vekhov, Maksim Saprykin, Maksim Shapovalov, Marina Levina, Boris Levin

**Affiliations:** 1 Papanin Institute for Biology of Inland Waters Russian Academy of Sciences, Borok, Russia Papanin Institute for Biology of Inland Waters Russian Academy of Sciences Borok Russia; 2 Azov-Black Sea branch of “VNIRO” (“AZNIIRKH”), Rostov-on-Don, Russia Azov-Black Sea branch of “VNIRO” (“AZNIIRKH”) Rostov-on-Don Russia; 3 Research Institute for Complex Problems at Adyghe State University, Maykop, Russia Research Institute for Complex Problems at Adyghe State University Maykop Russia; 4 Zoological Institute, Russian Academy of Sciences, Saint-Petersburg, Russia Zoological Institute, Russian Academy of Sciences Saint-Petersburg Russia; 5 Cherepovets State University, Cherepovets, Russia Cherepovets State University Cherepovets Russia

**Keywords:** fish fauna, database, Kuban River, Sea of Azov, Caucasus

## Abstract

**Background:**

This publication describes a dataset containing information on 1328 occurrences of fishes in the Kuban River Basin, the longest river of Northern Caucasus and representing its own freshwater ecoregion (428 Kuban Ecoregion). All observations have precise geo-referencing with the names of water bodies (rivers, lakes etc.). The dataset is based on both literature data (509 occurrences) and our own sampling (814 occurrences). Observations were carried out between 1889 and 2020.

**New information:**

The majority (> 61%) of occurrences in the presented data are published for the first time. This extended dataset contributes significantly to fish fauna survey in the Kuban River ecoregion.

## Introduction

**Figure 1.**
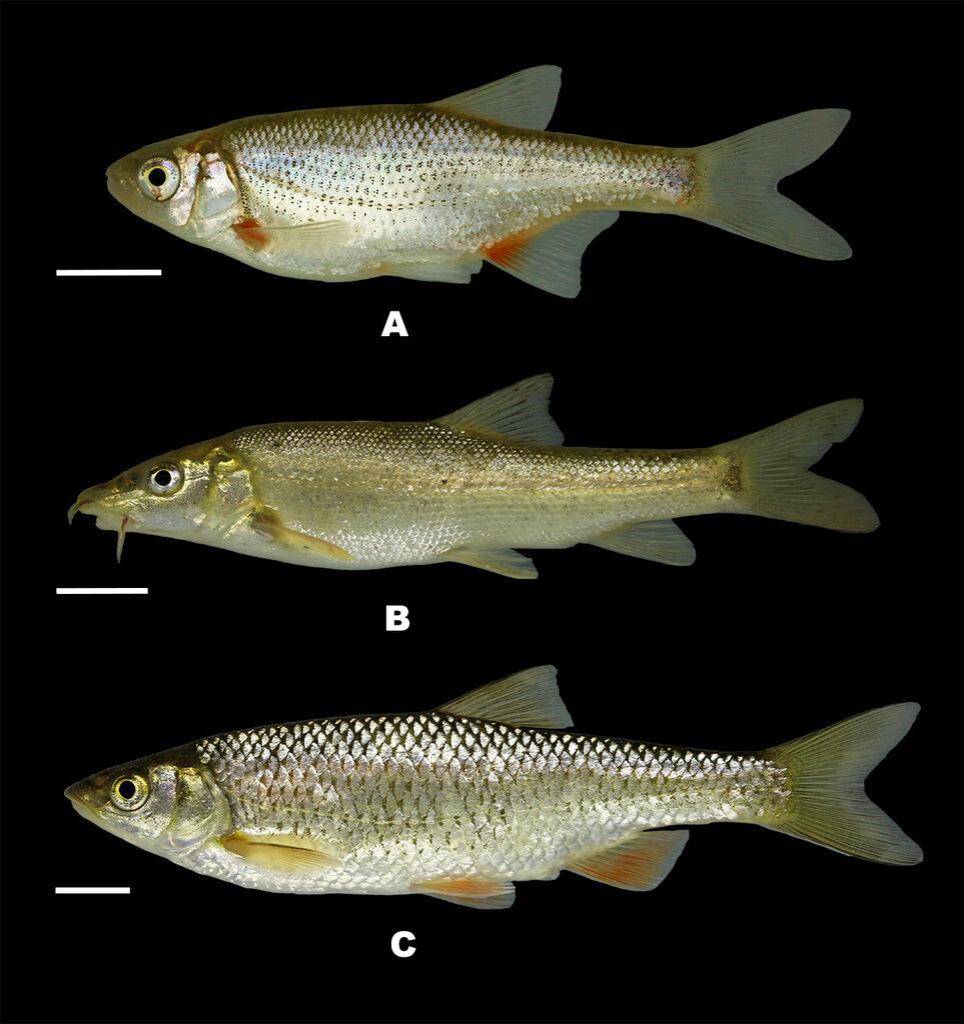
Photographs of some endemic fish species of the Kuban Basin: **A** *Alburnoideskubanicus* (Kudako River); **B** *Barbuskubanicus* (Ubin River); **С** *Petroleuciscusaphipsi* (Il' River). Scale bar - 1 cm. Photographs by Oleg Artaev.

Ichthyofauna of the Kuban River system is comprised of three groups: the species also inhabiting other European rivers, the species inhabiting brackish waters of the Azov Sea and migrated to the Kuban, as well as by the endemic species found only in the Kuban Basin. The fish fauna of the Kuban River Basin is one of the most distinct amongst river basins of European Russia. In total, 94 fish species have been recorded in the Kuban Basin (Bogutskaya and Naseka 2002, Abell et al. 2008, our data) including 11 endemic species and evolutionary significant units: *Alburnoideskubanicus* Bănărescu, 1964, *Barbuskubanicus* Berg, 1912, *Chondrostomakubanicum* Berg, 1914, Eudontomyzoncf.mariae (Berg, 1931), *Gobiokubanicus* Vasil'eva, 2004, *Petroleuciscusaphipsi* (Aleksandrov, 1927), *Phoxinus* sp., Ponticolacf.constructor (Nordmann, 1840), *Romanogobioparvus* Naseka et Freyhof, 2004, *R.pentatrichus* Naseka et Bogutskaya, 1998, *Sabanejewiakubanica* Vasil'eva & Vasil'ev, 1988 (Naseka 2010) (see photographs of some Kuban endemic fish species in Fig. 1). All endemic fish species are listed in the dataset presented.

The Middle Kuban River is considered an important place for the conservation of freshwater fishes in the Caucasus (Freyhof et al. 2020). The Kuban Basin is a separate ecoregion (428 in Abell et al. 2008), fish fauna of which is distinct from other ecoregions of the Caucasus. The native fauna is also clearly distinct from that of Don (ecoregion 427), which is geographically close, but has a different geological history (Bogutskaya and Hales 2021). In addition to new occurrence records, the dataset presented contains information on the type localities of the endemic species with data on museum numbers of type specimens. The basin of the Kuban River is located in Northern Caucasus, a highly populated region with developed agriculture. High anthropogenic activity resulted in numerous alien fish invasions, some of which are now naturalised. Most of the alien species are from East and South-eastern Asia [*Ctenopharyngodonidella* (Valenciennes, 1844); *Hypophthalmichthysmolitrix* (Valenciennes, 1844); *Hypophthalmichthysnobilis* (Richardson, 1845); *Oryziassinensis* Chen, Uwa & Chu, 1989; *Pseudorasboraparva* (Temminck & Schlegel, 1846)], North America [*Gambusiaholbrooki* Girard, 1859; *Ictaluruspunctatus* (Rafinesque, 1818); *Ictiobusbubalus* (Rafinesque, 1818); *I.cyprinellus* (Valenciennes, 1844); *I.niger* (Rafinesque, 1819); *Piaractusbrachypomus* (Cuvier, 1818); *Polyodonspathula* (Walbaum, 1792); *Rociooctofasciata* (Regan, 1903)] and a few species from Africa [*Oreochromisaureus* (Steindachner, 1864) and *O.mossambicus* (Peters, 1852)] (Moskul 1998, Moskul et al. 2012). The fish fauna of the Kuban Basin has been studied for a long time (e.g. Aleksandrov 1927, Berg 1949, Sukhanova and Troitskiy 1949, Tamanskaya and Troitskiy 1957, Troitskiy and Tsunikova 1988), but few publications contained data for certain localities. The goal of the study was to collect comprehensive data on occurrences of the Kuban fish species and to make these data available using GBIF (Artaev et al. 2021). The information on species distributions can be used by ichthyologists, ecologists, conservation biologists and managers of areas of nature protection.

## Sampling methods

### Study extent

The dataset contains information on 1328 occurrence records (one species in a definite place at a definite time) of 63 taxa, 58 of which were identified at species level, while six taxa were identified at generic level. The occurrences were recorded between 1889 and 2020. The study area is ~ 57900 km^2^.

### Sampling description

Occurrences retrieved from literature are based mainly on data from the fish elevator of the Krasnodar Reservoir (Akseleva 2017, Polin and Strelchenko 2018, Mischenko 2019, Polin and Strelchenko 2019). Our data are based on fish sampling using various fishing gear (frame net, seine net, gill net and cast net).

### Quality control

Each observation contains fundamental information, such as locality (coordinates), date, name of water body, name of observer and name of identifier. Geographical latitude/longitude coordinates for the majority of localities were obtained using hand-held GPS devices, while coordinates for localities extracted from literature and those missing coordinates were determined using the Google Maps service. Species were identified, based on morphological characters (Berg 1949, Bogutskaya and Poznyak 1994, Naseka and Bogutskaya 1998, Naseka and Freyhof 2004, Vasil'eva et al. 2004).

### Step description

First, we analysed published data on fish records. Second, we added our data on fish occurrences.

## Geographic coverage

### Description

**Figure 2.**
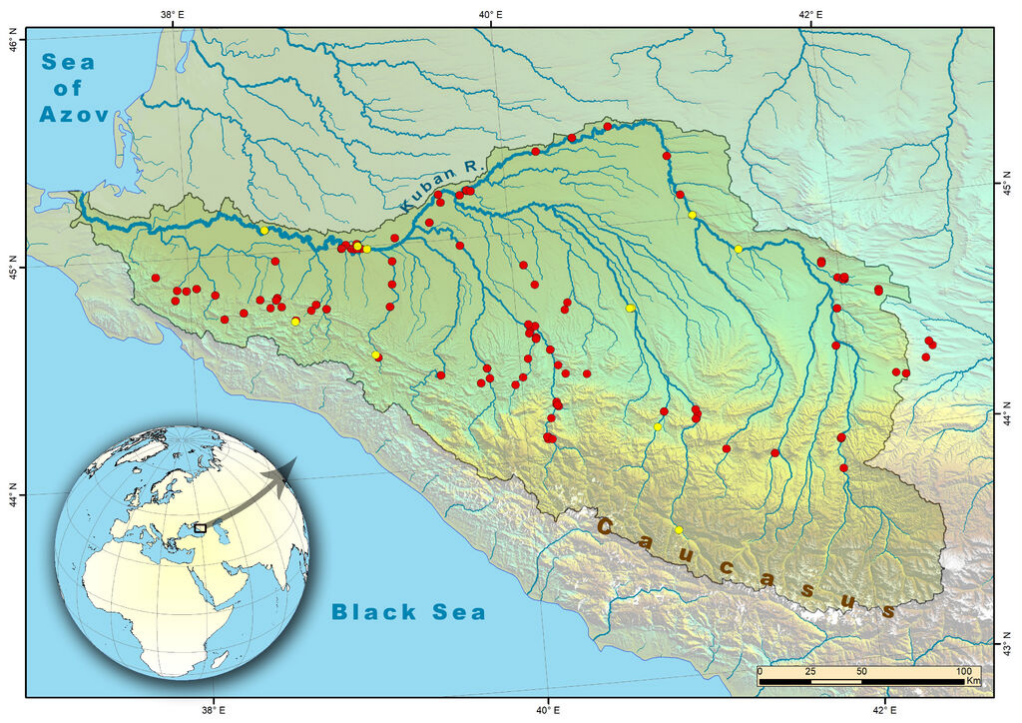
Map of sampling sites in the Kuban River Basin. Map was created in ArcGIS 10.8 software (www.esri.com). Author's data are designated by the red circles, while literature data are designated by yellow circles.

All occurrences were recorded within the Kuban River Basin which drains the North-western Caucasus and discharges into the Azov Sea and within the Large Stavropol' irrigation canal draining the the eastern part of the Kuban Basin and parts of the Kuma and Terek Rivers (both belong to Caspian Sea drainage – Fig. 2; the three most eastern localities out of the Kuban Basin belong to the Large Stavropol irrigation canal) and the upper part of Stavropol' canal discharging waters to the Manych-Don system. The length of the Kuban River is 870 km and watershed area is ca. 57900 km^2^. The Basin can be subdivided into three geographical zones: highlands, submontane and lowlands. The main drainage area is the northern slopes of the Caucasus with 2600 mm precipitation (Kupriyanov 1973). Lower reaches of the Kuban are located in agricultural landscapes and a significant volume of the Kuban water is taken for irrigation. There is one reservoir on the Kuban River, the Krasnodar Reservoir, located in its lower reach. This is the largest reservoir in the Northern Caucasus. It was built at 1973 with an area around 400 km^2^; its length is 46 km and 8-11 km wide (Pogorelov and Laguta 2019). Climate is mild in the middle and lower reaches of Kuban Basin with sub-zero daily temperature only during December-March (Kupriyanov 1973).

### Coordinates

43.23 and 45.76 Latitude; 36.77 and 42.33 Longitude.

## Taxonomic coverage

### Description

The dataset contains information on 63 taxa, of which 57 were identified at the species level and six at the genus level (Table 1). The species detected belong to 47 genera, 18 families and two classes. A few taxa need further commentaries on their taxonomic status. Individuals identified as *Rutilusrutilus* (Linnaeus, 1758) were replaced by *R.lacustris* (Pallas, 1814) according to results from genetic studies (Levin et al. 2017, Artaev et al. 2021). For a long time, the Kuban was thought to be home to only one *Barbus* species, endemic to the Kuban system, *B.kubanicus*. A recent genetic study (Levin et al. 2019) revealed that the upper reach of the Abin River, a left tributary of the Kuban River, is additionally inhabited by *B.tauricus* with hybridisation observed between these *Barbus* spp.

The connection of the Kuban system with the Kuma and Terek riverine systems via the Large Stavropol' irrigation canal, as well as with the Manych-Don system via the Nevinnomysk irrigation canal, may facilitate exchange of fish fauna as exemplified by *B.kubanicus* occurrences in the Manych system (Poznyak 1987). We consider the Prussian carp as *Carassiusauratus* (Linnaeus 1758) species complex since its taxonomic status is still under debate (Wouters et al. 2012, Rylková et al. 2013, Vekhov 2013, Šimková et al. 2015).

## Temporal coverage

### Notes

Data can be divided into three periods: i) 1889-1911 - data on the type localities of endemic species of the Kuban Basin; ii) 1974-2001 - data on the occurrences solely from literature sources; and iii) 2003-2020 - author's data and further literature data.

## Usage licence

### Usage licence

Creative Commons Public Domain Waiver (CC-Zero)

## Data resources

### Data package title

Fish occurrences in the Kuban River Basin

### Resource link

https://www.gbif.org/dataset/8d8218b1-835d-43ef-ac2d-34c746277528

### Alternative identifiers

https://doi.org/10.15468/82j8u8

### Number of data sets

1

### Data set 1.

#### Data set name

Fish occurrence in the Kuban River Basin

#### Data format

Darwin Core

#### Number of columns

30

#### Description

Dataset is a compilation of data as a contemporary faunistic research (2003-2020) and literature data (1974-1987) with indication of locality.

**Data set 1.** 

Column label	Column description
occurrenceID	The Globally Unique Identifier number for the recored.
basisOfRecord	The specific nature of the data record: HumanObservation.
eventDate	date format as YYYY-MM-DD.
scientificName	The full scientific name including the genus name and the lowest level of taxonomic rank with the authority.
kingdom	The full scientific name of the kingdom in which the taxon is classified.
phylum	The full scientific name of the phylum or division in which the taxon is classified.
class	The full scientific name of the class in which the taxon is classified.
order	The full scientific name of the order in which the taxon is classified.
family	The full scientific name of the family in which the taxon is classified.
decimalLatitude	The geographic latitude of location in decimal degrees.
decimalLongitude	The geographic longitude of location in decimal degrees.
Country	The name of the country (Russia).
countryCode	The standard code for the country in which the Location occurs.
individualCount	The number of individuals represented present at the time of the Occurrence.
year	Year of the event was recorded.
month	The month of the event was recorded.
day	The integer day of the month on which the Event occurred.
recordedBy	A person or group responsible for recording the original Occurrence.
identifiedBy	A list of names of people, who assigned the Taxon to the subject.
locality	The specific description of the place.
associatedReferences	Bibliographic reference of literature associated with the Occurrence.
coordinatePrecision	A decimal representation of the precision of the coordinates given in the decimalLatitude and decimalLongitude.
catalogNumber	An identifier (unique) for the record within the dataset or collection (only for type specimens).
occurrenceRemarks	Comments or notes about the Occurrence - what types of specimens were caught in this place.
identificationRemarks	Comments or notes about the Identification - what Latin name was given when describing a species from this locality.
institutionCode	The name (or acronym) in use by the institution having custody of the object(s) or information referred to in the record.
geodeticDatum	The ellipsoid, geodetic datum or spatial reference system (SRS) upon which the geographic coordinates given in decimalLatitude and decimalLongitude are based.
identificationQualifier	A brief phrase or a standard term ("cf.", "aff.") to express the determiner's doubts about the Identification.
taxonRank	The taxonomic rank of the most specific name in the scientificName.
taxonRemarks	Comments or notes about the taxon or name.

## Figures and Tables

**Table 1.** Occurrences of fish taxa in the Kuban Basin represented in the dataset.

**Taxa**	**Number of occurrences**
Acipenseridae	
*Acipensergueldenstaedtii* Brandt & Ratzeburg, 1833	1
*Acipensernudiventris* Lovetsky, 1828	1
*Acipenserruthenus* Linnaeus, 1758	21
*Acipenserstellatus* Pallas, 1771	6
*Husohuso* (Linnaeus, 1758)	1
Cichlidae	
*Oreochromisaureus* (Steindachner, 1864)	4
*Rociooctofasciata* (Regan, 1903)	3
Clupeidae	
*Alosaimmaculata* Bennett, 1835	8
*Alosamaeotica* (Grimm, 1901)	7
*Alosatanaica* (Grimm, 1901)	17
Cobitidae	
*Sabanejewiakubanica* Vasil'eva & Vasil'ev, 1988	30
Cyprinidae	
*Abramisbrama* (Linnaeus, 1758)	53
*Alburnoideskubanicus* Banarescu, 1964	85
*Alburnusalburnus* (Linnaeus, 1758)	73
*Alburnusleobergi* Freyhof & Kottelat, 2007	37
*Barbus* Daudin, 1805	16
*Barbuskubanicus* Berg, 1912	47
*Bliccabjoerkna* (Linnaeus, 1758)	39
*Carassiusauratus* (Linnaeus, 1758)	20
*Carassius* Jarocki, 1822	31
*Chondrostomakubanicum* Berg, 1914	12
*Ctenopharyngodonidella* (Valenciennes, 1844)	18
*Cyprinuscarpio* Linnaeus, 1758	33
*Gobiocaucasicus* Kamensky, 1901	21
*Gobio* Cuvier, 1816	67
*Gobioholurus* Fowler, 1976	1
*Gobiokubanicus* Vasil'eva, 2004	1
*Leucaspiusdelineatus* (Heckel, 1843)	3
*Leuciscusaspius* (Linnaeus, 1758)	32
*Leuciscusidus* (Linnaeus, 1758)	1
*Pelecuscultratus* (Linnaeus, 1758)	35
*Petroleuciscusaphipsi* (Aleksandrov, 1927)	45
*Petroleuciscusborysthenicus* (Kessler, 1859)	1
*Phoxinus* Rafinesque, 1820	17
*Pseudorasboraparva* (Temminck & Schlegel, 1846)	7
*Rhodeusamarus* (Bloch, 1782)	17
*Romanogobioparvus* Naseka & Freyhof, 2004	1
*Romanogobiopentatrichus* Naseka & Bogutskaya, 1998	2
*Rutiluslacustris* (Pallas, 1814)	87
*Scardiniuserythrophthalmus* (Linnaeus, 1758)	13
*Squaliuscephalus* (Linnaeus, 1758)	71
*Vimbavimba* (Linnaeus, 1758)	29
Esocidae	
*Esoxlucius* Linnaeus, 1758	17
Gasterosteidae	
*Pungitiusplatygaster* (Kessler, 1859)	3
Gobiidae	
*Knipowitschia* Iljin, 1927	1
*Neogobiusfluviatilis* (Pallas, 1814)	31
*Pomatoschistus* Gill, 1863	2
Ponticolacf.constructor (Nordmann, 1840)	9
*Proterorhinussemipellucidus* (Kessler, 1877)	4
*Proterorhinus* Smitt, 1900	13
Ictaluridae	
*Ictaluruspunctatus* (Rafinesque, 1818)	15
Mugilidae	
*Planilizahaematocheila* (Temminck & Schlegel, 1845)	2
Nemacheilidae	
*Barbatulabarbatula* (Linnaeus, 1758)	28
Percidae	
*Gymnocephaluscernua* (Linnaeus, 1758)	21
*Percafluviatilis* Linnaeus, 1758	48
*Sanderlucioperca* (Linnaeus, 1758)	57
*Sandervolgensis* (Gmelin, 1789)	5
Petromyzontidae	
Eudontomyzoncf.mariae (Berg, 1931)	1
Poeciliidae	
*Gambusiaholbrooki* Girard, 1859	6
Salmonidae	
*Salmolabrax* Pallas, 1814	4
Serrasalmidae	
*Piaractusbrachypomus* (Cuvier, 1818)	1
Siluridae	
*Silurusglanis* Linnaeus, 1758	31
Syngnathidae	
*Syngnathusabaster* Risso, 1827	15

## References

[B7434814] Abell Robin, Thieme Michele L., Revenga Carmen, Bryer Mark, Kottelat Maurice, Bogutskaya Nina, Coad Brian, Mandrak Nick, Balderas Salvador Contreras, Bussing William, Stiassny Melanie L. J., Skelton Paul, Allen Gerald R., Unmack Peter, Naseka Alexander, Ng Rebecca, Sindorf Nikolai, Robertson James, Armijo Eric, Higgins Jonathan V., Heibel Thomas J., Wikramanayake Eric, Olson David, López Hugo L., Reis Roberto E., Lundberg John G., Sabaj Pérez Mark H., Petry Paulo (2008). Freshwater ecoregions of the world: A new map of biogeographic units for freshwater biodiversity conservation. BioScience.

[B7075296] Akseleva Yu. Yu., Pavlov S. I. (2017). Population dynamics and fish species composition of the fish-passing construction of Fedorov dam from 2011 to 2015. Bioecological study of local lore: world, Russian, regional problems.

[B7444063] Aleksandrov A. I. (1927). Materials on the fauna of the Kuban River Basin. Proceedings of the Kerch Ichthyological Laboratory.

[B7476337] Artaev O, Pashkov A, Vekhov D, Shapovalov M, Saprykin M, Levin B (2021). Fish occurrence in Kuban River Basin. https://www.gbif.org/dataset/8d8218b1-835d-43ef-ac2d-34c746277528.

[B7075184] Artaev O. N., Ermakov O. A., Vekhov D. A., Konovalov A. F., Levina M. A., Pozdeev I. V., Ruchin A. B., Alyushin I. V., Iljin V. Yu., Levin B. A. (2021). Genetic screening of distribution pattern of roaches *Rutilusrutilus* and *R.lacustris* (Cyprinidae) in broad range of secondary contact (Volga Basin). Inland Water Biology.

[B7549704] Berg L. S. (1949). Рыбы пресных вод СССР и сопредельных стран.

[B7549678] Bogutskaya Nina, Hales Jennifer FEOW. https://www.feow.org/ecoregions/details/428.

[B7568842] Bogutskaya N. G., Poznyak V. G. (1994). Redescription of *Leuciscusaphipsi* Aleksandrov (Leuciscinae, Cyprinidae). Voprosy Ikhtiologii.

[B6947976] Bogutskaya N. G., Naseka A. M. (2002). Freshwater fish species list. Kuban. https://www.zin.ru/animalia/pisces/rus/taxbase_r/fauna_r/srchresultreg_r.asp?region=K.

[B7444045] Freyhof J., Pipoyan S., Mustafayev N., brahimov S., Japoshvili B., Sedighi O., Levin B., Pashkov A, Turan D., Zazanashvili N., Garforth M., Bitsadze M. (2020). Ecoregional Conservation Plan for the Caucasus, 2020 Edition: Supplementary Reports. WWF, KfW, Tbilisi. 97-105 pp.

[B7070050] Kupriyanov V. V. (1973). Ресурсы поверхностных вод СССР.

[B7075091] Levin Boris A., Gandlin Alexander A., Simonov Evgeniy S., Levina Marina A., Barmintseva Anna E., Japoshvili Bella, Mugue Nikolai S., Mumladze Levan, Mustafayev N. J., Pashkov Andrey N., Roubenyan Haikaz R., Shapovalov Maxim I., Doadrio Ignacio (2019). Phylogeny, phylogeography and hybridization of Caucasian barbels of the genus *Barbus* (Actinopterygii, Cyprinidae). Molecular Phylogenetics and Evolution.

[B7075164] Levin B. A., Simonov E. P., Ermakov O. A., Levina M. A., Interesova E. A., Kovalchuk O. M., Malinina Y. A., Mamilov N. S., Mustafayev N. J., Pilin D. V., Pozdeev I. V., Prostakov N. I., Roubenyan H. R., Titov S. V., Vekhov D. A. (2017). Phylogeny and phylogeography of the roaches, genus *Rutilus* (Cyprinidae), at the Eastern part of its range as inferred from mtDNA analysis. Hydrobiologia.

[B7075310] Mischenko M. V., Mamas' N. N. (2019). Reduction of the number, species diversity of fisches in river Kuban. Ecology of river landscapes.

[B7450025] Moskul G. A. (1998). Fish of reservoirs of the Kuban Basin (key).

[B7450016] Moskul G. A., Kovalenko Yu. I., Pashinova N. G., Bolkunov O. A. (2012). Current status and prospects for fisheries use of the Azov-Kubanian limans.

[B7568833] Naseka A. M., Bogutskaya N. G. (1998). A new gudgeon species *Romanogobiopentatrichus* (Gobioninae, Cyprinidae) from the basin of the Kuban River. Voprosy Ikhtiologii.

[B7568824] Naseka A. M., Freyhof J. (2004). Romanogobioparvus, a new gudgeon from River Kuban, southern Russia (Cyprinidae, Gobioninae). Ichthyological Exploration of Freshwaters.

[B7476328] Naseka A. M. (2010). Zoogeographical freshwater divisions of the Caucasus as a part of the West Asian Transitional Region. Proceedings of the Zoological Institute RAS.

[B7070701] Pogorelov A. V., Laguta A. A. (2019). Krasnodar Reservoir: state and transformation during the period of exploitation [Краснодарское водохранилище: состояние и трансформация за период эксплуатации]. Regional Geographic Research [Региональные географические исследования].

[B7075282] Polin A. A., Strelchenko O. V., Bogachev A. N., Belousov V. N., Kornienko G. G., Boyko N. E. (2018). Species composition, dynamics of the fish population, fish ladder transplanted Krasnodar Reservoir in 2017. Topical issues of fishing, fish farming (aquaculture), ecological monitoring of aquatic ecosystems. Materials of the International Scientific, Practical Conference dedicated to the 90th anniversary of the Azov Research Institute of Fisheries.

[B7075259] Polin A. A., Strelchenko O. V., Belousov V. N. (2019). Species composition, dynamics of the fish population, fish ladder transplanted Krasnodar Reservoir in 2018. Proceedings of AzNIIRKH.

[B7468894] Poznyak V. G. (1987). Fauna of Kalmykia. Fishes.

[B7433016] Rylková Kateřina, Kalous Lukáš, Bohlen Jörg, Lamatsch Dunja K., Petrtýl Miloslav (2013). Phylogeny and biogeographic history of the cyprinid fish genus *Carassius* (Teleostei: Cyprinidae) with focus on natural and anthropogenic arrivals in Europe. Aquaculture.

[B7433007] Šimková Andrea, Hyršl Pavel, Halačka Karel, Vetešník Lukáš (2015). Physiological and condition-related traits in the gynogenetic-sexual *Carassiusauratus* complex: different investments promoting the coexistence of two reproductive forms?. BMC Evolutionary Biology.

[B7075081] Sukhanova E. R., Troitskiy S. K. (1949). Ichthyofauna at the sites of spawning of Vimba bream and Bleak in Psecups river. Proceedings of the fish-biological laboratory AZCHERRYBVOD.

[B7075063] Tamanskaya G. G., Troitskiy S. K. (1957). Ichthyofauna and fishery significance of the Belaya River (Kuban River Basin). Proceedings of Fish-Breeding Biological Laboratory of AZCHERGOSRYBVOD.

[B7074995] Troitskiy S. K., Tsunikova E. P. (1988). Рыбы бассейнов нижнего Дона и Кубани.

[B7568851] Vasil'eva E. D., Vasil'ev V. P., Kuga T. I. (2004). On taxonomy of gudgeons of the genus *Gobio* (Gobioninae, Cyprinidae) of Europe: a new species of gudgeon *Gobiokubanicus* sp. nova from the Kuban River Basin. Voprosy Ikhtiologii.

[B7075223] Vekhov D. A. (2013). Some problematic issues of biology goldfish *Carassiusauratus* s. lato. Scientific and Technical Bulletin of Laboratory of Ichthyology INENKO.

[B7432998] Wouters J., Janson S., Lusková V., Olsén K. H. (2012). Molecular identification of hybrids of the invasive gibel carp *Carassiusauratusgibelio* and crucian carp *Carassiuscarassius* in Swedish waters. Journal of Fish Biology.

